# Attenuated androgen discontinuation in patients with hereditary angioedema: a commented case series

**DOI:** 10.1186/s13223-021-00644-0

**Published:** 2022-01-13

**Authors:** Marcus Maurer, Markus Magerl, Emel Aygören-Pürsün, Konrad Bork, Henriette Farkas, Hilary Longhurst, Sorena Kiani‑Alikhan, Laurence Bouillet, Isabelle Boccon-Gibod, Mauro Cancian, Andrea Zanichelli, David Launay

**Affiliations:** 1 Department of Dermatology and Allergy, Charité–Universitätsmedizin Berlin, Angioedema Center of Reference and Excellence (ACARE), Dermatological Allergology, Allergie-Centrum-Charité, Berlin, Germany; 2 Goethe University Frankfurt, Frankfurt, Germany; 3 Department of Dermatology, Johannes Gutenberg University, Mainz, Germany; 4 Hungarian Angioedema Center of Excellence and Reference (ACARE), Department of Internal Medicine and Haematology, Semmelweis University, Budapest, Hungary; 5 Auckland District Health Board and University of Auckland, Auckland, New Zealand; 6 Angioedema Center of Reference and Excellence (ACARE), Barts Health NHS Trust, London, UK; 7 French National Center of Reference and Excellence for Angioedema, Grenoble Alpes University Hospital, Grenoble, France; 8 Italian Network for Hereditary and Acquired Angioedema (ITACA), Interregional Center of Reference for Angioedema, University of Padova, Padova, Italy; 9 Italian National Center of Reference for Angiodema, Department of Internal Medicine, ASST Fatebenefratelli Sacco, Ospedale Luigi Sacco – Università degli Studi di Milano, Milan, Italy; 10 University of Lille, Inserm, CHU Lille, U1286 - INFINITE - Institute for Translational Research in Inflammation, French National Center of Reference for Angioedema, 59000 Lille, France

**Keywords:** Angioedema, hereditary, Prophylaxis, Attenuated androgens, Danazol, Oxandrolone, Case series

## Abstract

**Background:**

Hereditary angioedema (HAE) is characterized by potentially severe and life-threatening attacks of localized swelling. Prophylactic therapies are available, including attenuated androgens. Efficacy of attenuated androgens has not been assessed in large, randomized, placebo-controlled trials and can be associated with frequent, and sometimes severe, side effects. As better tolerated targeted therapies become available, attenuated androgen withdrawal is increasingly considered by physicians and their patients with HAE. Attenuated androgens withdrawal has not been systematically studied in HAE, although examination of other disorders indicates that attenuated androgen withdrawal may result in mood disturbances and flu-like symptoms. Standardized protocols for attenuated androgen discontinuation that continue to provide control of attacks while limiting potential attenuated androgen withdrawal symptoms are not established as the outcomes of different withdrawal strategies have not been compared. We aim to describe the challenges of attenuated androgen discontinuation in patients with HAE and how these may continue into the post-androgen period.

**Case presentation:**

We present a retrospective case series of 10 patients with confirmed type I HAE who have discontinued prophylactic treatment with attenuated androgens. The most common reason for attenuated androgen discontinuation was side effects. Attenuated androgens were either immediately withdrawn, tapered and/or overlapped with another treatment. The major challenge of discontinuation was the management of an increased frequency and severity of HAE attacks in some patients.

**Conclusions:**

Healthcare teams need to undertake careful planning and monitoring after attenuated androgens discontinuation, and modify treatment strategies if HAE control is destabilized with an increased number of attacks. Discontinuation of attenuated androgens is definitively an option in an evolving HAE treatment landscape, and outcomes can be favourable with additional patient support and education.

**Supplementary Information:**

The online version contains supplementary material available at 10.1186/s13223-021-00644-0.

## Background

Hereditary angioedema (HAE) is an inherited disorder characterized by unpredictable attacks of localized swelling in the skin and/or mucosa [1, 2]. HAE is most frequently caused by mutations in *SERPING1*, resulting in reduced production (type I HAE) or dysfunction (type II HAE) of the C1-inhibitor (C1-INH) protein. This leads to vasodilation, increased capillary permeability and swelling, mediated by bradykinin (which is generated by the contact activation system) [1, 3–5]. HAE attacks are recurrent and frequently affect the face, extremities, upper airway and abdomen [2, 4]. Even mild attacks may cause transient discomfort and disfigurement, whereas abdominal attacks can be so painful that they may mimic an acute abdomen (leading to inappropriate surgery), and swelling of the larynx can be fatal [1, 2]. Because of the unpredictability and potential severity of HAE attacks, it is recommended that all patients are evaluated for the need for long-term prophylactic treatments [6]. Several prophylactic therapies are available (Table 1), which either replace deficient C1-INH or inhibit kallikrein – a component of the contact system that catalyses the production of bradykinin. An additional and historical option to targeted therapies for long-term prophylaxis is attenuated androgen (AA) treatment. AAs, such as danazol, stanozolol and oxandrolone, have not been studied in large, randomized, placebo-controlled trials, and available data are from limited numbers of patients [11, 19–21]. Treatment effects can be highly variable, and although some studies support the efficacy of androgens [11, 19], others show suboptimal outcomes [22, 23]. AAs are associated with side effects in approximately 80% of patients in some studies [6, 19, 20, 24]. As outlined in Table 1, these side effects may take a variety of forms including, but not limited to, weight gain, hypertension, proatherogenic lipid profile changes, headaches, cramps, mood disturbances (such as depression and anxiety), acne, and polycythaemia [11, 21, 24–27]. Although the safety profiles of AAs are derived from studies in small numbers of patients, with the potential for the majority of treated patients to be affected, the use of AAs and patient monitoring must be carefully evaluated. Further, AAs may not be appropriate in female patients because of potential virilisation and menstrual irregularities, and are contraindicated during pregnancy because of possible virilisation of female fetuses [6, 21, 24–26, 28]. In children and adolescents, AA use is not appropriate because of potential effects on bone development [6, 29], and the potential risk of early puberty [30, 31]. AAs are contraindicated in several conditions such as cardiovascular diseases or cancer, and also with a large number of drugs [18]. As effective and better tolerated targeted options for long-term prophylaxis are approved or developed [32–37], the use of AAs is decreasing and AA discontinuation is becoming an increasingly used option or necessity because of side effects, contraindications and/or patient/physician preference. Although other treatment options may be preferred to androgens, the higher cost of the former may be a hurdle to their use in some countries and regions [36]. However, a US study in 2015 indicated that the proportion of physicians who specified a preference for long-term prophylaxis with danazol decreased from 56 to 23% between 2010 and 2013 [38, 39].

**Table 1:** Prophylactic treatments for HAE

Drug name	Mode of action	Administration	Self-administration	Safety events^a,b^
Plasma-derived C1-INH (pdC1-INH) [7]	C1-INH replacement	Intravenous (IV)	Yes	*Very common*: headache; nausea *Common*: hypersensitivity; dizziness; vomiting; rash, erythema, pruritus; injection site rash/erythema, infusion site pain, pyrexia *Uncommon*: hyperglycaemia; venous thrombosis, phlebitis, venous burning, hot flush; cough; diarrhoea, abdominal pain; contact dermatitis; joint swelling, arthralgia, myalgia; chest discomfort
pdC1-INH [8]	C1-INH replacement	Subcutaneous (SC)	Yes	*Rare*: development of thrombosis; rise in temperature, injection site reactions; allergic or anaphylactic-type reactions
Lanadelumab [9]	Plasma kallikrein inhibition	Subcutaneous (SC)	Yes	*Very common*: injection site reactions *Common*: hypersensitivity; dizziness; maculopapular rash; myalgia; increased alanine aminotransferase, increased aspartate aminotransferase
Berotralstat [10]	Plasma kallikrein inhibition	Oral	Yes	*Very common*: headache; abdominal pain, diarrhoea *Common:* vomiting, gastroesophageal reflux, flatulence; rash; alanine aminotransferase increases, aspartate aminotransferase increases
Attenuated androgens^c^ (danazol, oxandrolone) [11–18]	Unknown, but potentially through increased C1‑INH levels and/or metabolism of bradykinin	Oral	Yes	*Blood and lymphatic disorders*: increase in red cell and platelet count; reversible polycythaemia, leucopenia, thrombocytopenia, eosinophilia, and splenic peliosis *Endocrine disorders*: androgenic effects (acne, weight gain, increased appetite, seborrhoea, hirsutism, hair loss, voice change, which may take the form of hoarseness, sore throat or instability or deepening of pitch; hypertrophy of the clitoris, fluid retention); other endocrine effects (menstrual disturbances in the form of spotting, alteration of the timing of the cycle and amenorrhoea; flushing, vaginal dryness, changes in libido, vaginal irritation and reduction in breast size; modest reduction in spermatogenesis) *Metabolic and nutrition disorders*: increased insulin resistance, increase in plasma glucagon, mild impairment of glucose tolerance; increase in low-density lipoprotein cholesterol, decrease in high-density lipoprotein cholesterol, affecting all subfractions, and decrease in apoliproteins AI and AII; induction of aminolevulinic acid synthetase, and reduction in thyroid binding globulin, T4, with increased uptake of T3, but without disturbance of thyroid stimulating hormone or free levothyroxine index *Psychiatric disorders*: emotional lability, anxiety, depressed mood and nervousness *Nervous system disorders*: dizziness, headache, vertigo, benign intracranial hypertension, migraine; aggravation of epilepsy, carpal tunnel syndrome *Eye disorders*: visual disturbances, such as blurring of vision, difficulty in focusing, difficulty in wearing contact lenses and refraction disorders requiring correction *Respiratory, thoracic and mediastinal disorders*: pleuritic pain, interstitial pneumonitis *Gastrointestinal disorders*: nausea, epigastric pain *Cardiac disorders*: hypertension, palpitations and tachycardia; thrombotic events, including saggital sinus, cerebrovascular thrombosis as well as arterial thrombosis; myocardial infarction *Hepatobiliary disorders*: isolated increases in serum transaminase levels, cholestatic jaundice, benign hepatic adenomata and pancreatitis; peliosis hepatitis as well as malignant hepatic tumour observed with long term use; hepatocellular injury, hepatic failure, jaundice hepatocellular, hepatocellular focal nodular hyperplasia *Skin and subcutaneous tissue disorders*: rashes, which may be maculopapular, petechial or purpuric and may be accompanied by fever, or may take an urticarial form and may be accompanied by facial oedema; sun-sensitive rash; inflammatory erythematosus nodules, changes in skin pigmentation, exfoliative dermatitis and erythema multiforme *Musculoskeletal and connective tissue disorders*: backache and muscle cramps, which can be severe, with elevation of creatine phosphokinase levels; muscle tremors, fasciculation, limb pain, joint pain and joint swelling *Renal and urinary disorders*: haematuria with prolonged use in patients with HAE *General disorders and administration site condition*: fatigue

^a^ Listed as in summary of product characteristics (SPC)

^b^ Frequencies of events are categorized as: Very common (≥ 1/10); Common (≥ 1/100 – < 1/10); Uncommon (≥ 1/1000 to  < 1/100); Rare (≥ 1/10,000 – < 1/1000); Very rare (< 1/10,000) [7–10]

^c^ Event frequency not categorized in SPC [18]

AA discontinuation can result in destabilization of control of HAE attacks on one side, and a withdrawal syndrome on the other, with mood disturbances, anxiety, depression, insomnia, fatigue, hypersomnia and a flu-like syndrome, although some of these symptoms have only been studied in populations receiving high doses of androgens [40–45]. Studies of AA withdrawal in HAE have not been extensively conducted. A survey of 12 physicians treating HAE has shown that physicians had patients who had experienced complications and/or side effects of AA discontinuation including fatigue and mood disturbances [45]. Surveyed physicians were also concerned with the potential for changes to attack rates. While potential strategies for AA withdrawal in HAE—tapering, overlapping with other therapies prior to tapering or stopping, and immediate switching—were suggested based on this physician survey and the broader literature on the use of endocrine treatments [45], these strategies have not yet been systematically compared in terms of patient outcomes and further work is needed to understand the impact of different strategies. Through this case series, we describe AA discontinuation in patients with HAE caused by C1-INH deficiency. We examine the challenges associated with AA discontinuation, present patient outcomes, and describe how treatment strategies need to be modified following AA discontinuation in order to further understanding of this topic.

## Case series

### Methods

An advisory board of leading European experts in HAE was convened to discuss AA discontinuation in patients with HAE, a topic of current interest because of the expanding landscape of targeted prophylactics for HAE. The experts agreed that a case series could highlight challenges of AA discontinuation to healthcare professionals who treat HAE, and raise considerations for how to manage the transition to alternative treatments. This review is a retrospective case series of patients with confirmed type I HAE who have discontinued or attempted to discontinue prophylactic treatment with AAs. Descriptive statistics only are provided. Written informed consent for publication has been provided by all patients, except for one patient who was deceased and for whom consent has been provided by next of kin. All patient data are anonymized, and direct identifiers are not included [46].

### Patient characteristics

The cases of 10 patients with confirmed type I HAE who either discontinued or are discontinuing AAs are presented. More comprehensive case details are provided as an Additional file 1. Three patients were female; the age range was between 31 and 76 years (median = 51 years). Patient characteristics and details of AA doses are shown in Table 2. The most commonly used AA was danazol (*n* = 8). Prior to AA discontinuation, all patients underwent AA dose modifications or a change of AA type (Table 2 and supplementary information). Time on AAs prior to discontinuation ranged from 1.5 to 36 years (median = 16.5 years).

**Table 2:** Patient characteristics, AA treatment, reasons for discontinuation and discontinuation strategy

Case	Sex	Age, years	AA	Dose prior to discontinuation	Time on AAs prior to discontinuation, years	Reason for discontinuation	Discontinuation strategy
1	Female	50	Danazol	200 mg QD^a^	28	Side effects at high doses and insufficient control of HAE attacks at lower dose ⦁ Headaches ⦁ Hypertension ⦁ Muscle cramps ⦁ Virilisation ⦁ Weight gain ⦁ Severe breakthrough attacks at lower AA doses	Immediate withdrawal
2	Male	34	Oxandrolone	5 mg QD^b^	1.5	Side effects and insufficient control of HAE attacks ⦁ Polycythaemia	Immediate withdrawal
3	Male	52	Danazol	200 mg QD^a^	26	Side effects ⦁ Headaches ⦁ Hypertension ⦁ Myalgia ⦁ Weight gain	Reduced to 100 mg QD for 2 weeks, then 100 mg QOD for 2 weeks, and finally 100 mg/week for 2 weeks, at the same time as 1,000 U pdC1-INH twice/week was introduced
4	Male	76	Danazol	300 mg QD^a^	18	Side effects and contraindications ⦁ Treated with angiotensin converting enzyme (ACE) inhibitors and statins, the latter of which resulted in rhabdomyolysis and acute kidney failure ⦁ Hypertension and high blood cholesterol	Immediate withdrawal
5	Female	64	Danazol	150 mg QD^c^	14	Contraindications ⦁ Treatment required for hormone-sensitive breast cancer ⦁ Surgery, radiotherapy and exemestane	Immediate withdrawal
6	Male	31	Danazol	200 mg five times/week^a^	13	Insufficient control of HAE attacks	Maintain danazol 200 mg five times/week for 2 weeks during the introduction of lanadelumab 300 mg every 14 days
7	Male	59	Danazol	100 mg QD^a^	9	Improved control of HAE attacks and side effects ⦁ Hypercholesterolaemia ⦁ Transaminase elevations, ⦁ Steatosis ⦁ Multifocal leukoencephalopathy	Immediate withdrawal
8	Male	48	Oxandrolone^d^	5 mg QD	15	Participation in a clinical trial	Immediate withdrawal (2 weeks prior to screening visit for study)
9	Female	43	Danazol	100 mg QOD^a^	29	Unplanned pregnancy	Immediate withdrawal
10	Male	62	Danazol	100 mg QD^a^	36	Loss of access to androgens	Reduced to 100 mg QOD for 1 week, then 100 mg/3 days for 3 weeks

QD: every day; QOD: every other day

^a^ Danazol dose modifications made to manage breakthrough attacks and/or identify the minimal effective dose

^b^ Starting dose of 5 mg QD was increased to 7.5 mg because abdominal attacks occurred every 2 weeks. Oxandrolone was stopped for 3 months because of polycythaemia and was reintroduced at 5 mg QD after resolution of this side effect

^c^ Reduced from 600 mg QD when the menopause started

^d^ Danazol not tolerated because of mood disturbances

### Reasons for AA discontinuation and methods of AA discontinuation

The most common reason for AA discontinuation was the occurrence of side effects (*n* = 5; Table 2). Side effects included headaches, hypertension and weight gain, among others. Insufficient control of HAE attacks affected the decision to discontinue AAs in 3 patients, and 1 patient was assessed as no longer requiring prophylaxis. Contraindications were responsible for discontinuation in a further 2 patients, while an unplanned pregnancy, participation in a clinical trial and loss of access to medication were other reasons for treatment switches (2 patients experienced side effects and insufficient control of HAE attacks; 1 patient experienced side effects and had no ongoing need for prophylaxis). In 7 patients, AAs were discontinued immediately with no gradual dose reductions. Of the remaining 3 patients, 2 decreased danazol gradually while a targeted therapy (lanadelumab or pdC1-INH) was introduced and 1 discontinued gradually.

### Control of HAE attacks after AA discontinuation

**Fig. 1:**
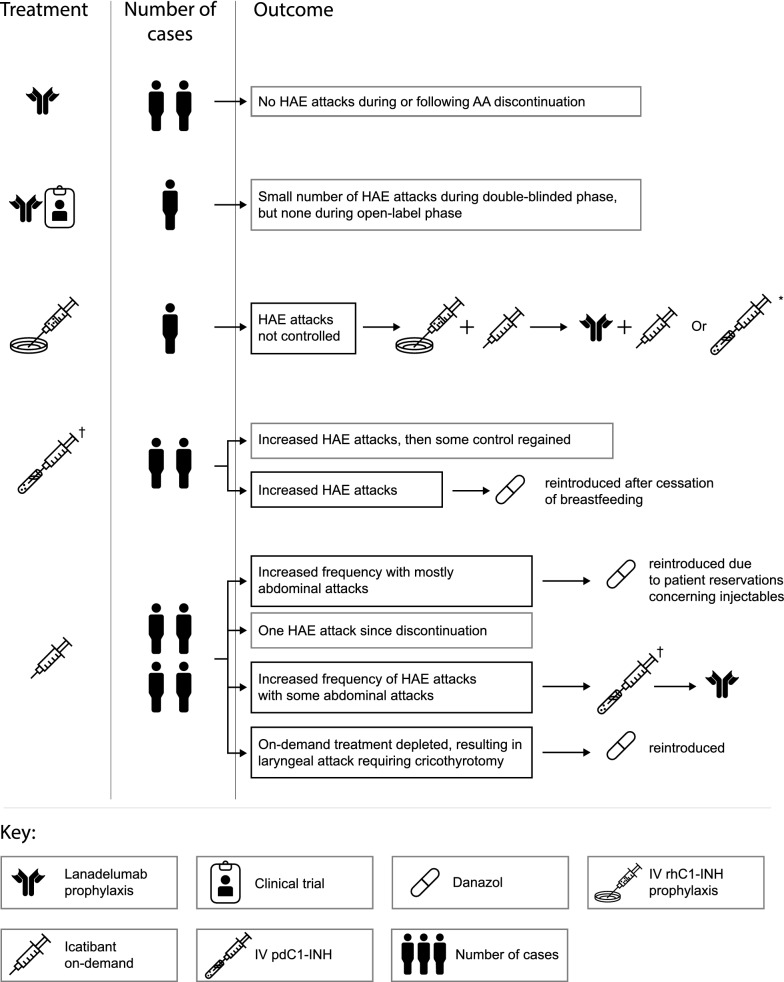
Initial treatments after attenuated androgen discontinuation, and patient outcomes. Patients were provided with a range of treatments, including prophylactic and on-demand options. In several patients, HAE attacks were not adequately controlled and further treatments were introduced. *On-demand; ^†^prophylaxis; IV, intravenous.

Outcomes of AA discontinuation are summarized in Fig. 1 and described for individual cases in Table 3.

**Table 3:** Outcomes of discontinuation

Case	Changes to HAE attack frequency and/or severity	Side effects during or after discontinuation	Replacement treatment for AAs	Management of HAE attacks and side effects	Time since discontinuation, months	Current patient outcome
1	No attacks	None	Lanadelumab 300 mg every 14 days	NA	10	No attacks (Angioedema Activity Score) High QoL (AE-QoL Questionnaire score) Headaches reduced and weight loss
2	Increased frequency to up to four attacks/week	None	rhC1-INH 12,600 U once/week	After 3 months, rhC1-INH was switched to IV pdC1-INH 2,000 U once/week with on-demand icatibant Breakthrough attacks continued and prophylactic lanadelumab 300 mg twice every 14 days was introduced, with on-demand icatibant 30 mg and/or pdC1-INH 2,000 U	26	No attacks QoL improved (clinician reported)
3	Increased frequency, with severe abdominal attacks	High weight	IV pdC1-INH 1,000 U twice/week	HAE attacks became milder and less frequent over a 2-month period	72	Zero to one attacks/year Good QoL (clinician reported) No reported hypertension, myalgia or headaches, and weight decreased
4	One attack in 7 years	None	Icatibant 30 mg on-demand	–	84	One attack in this time QoL has been affected by a stroke and other health conditions
5	Increased frequency, with abdominal attacks	Depression and anxiety, likely due to both cancer and HAE attacks	Icatibant 30 mg on-demand	Antidepressants, and IV prophylactic pdC1-INH 1,000 U/3 days introduced after 6 months Patient then switched to lanadelumab 300 mg every 14 days because of a deep vein thrombosis	84	No attacks Satisfied with prophylaxis but concerned with cancer progression
6	No attacks	None	Lanadelumab 300 mg every 14 days	None required	7	No attacks QoL improved (clinician reported)
7	Increased frequency and severity, with severe laryngeal attack	None	Icatibant 30 mg on-demand	Patient supply of on-demand therapy exhausted prior to laryngeal attack. The patient experienced respiratory failure. Cricothyrotomy and pdC1-INH 2,000 U were required, and danazol was reintroduced at 200 mg QD. Danazol was then tapered to 100 mg QD, and then 50 mg QD	NA	Non-optimal level of attacks Poor QoL (clinician reported)
8	One to two/month during double-blind phase of trial None on open-label lanadelumab 300 mg every 14 days	None	Placebo/lanadelumab during double-blind phase of trial Open-label lanadelumab 300 mg every 14 days	On-demand C1-INH for breakthrough attacks during double-blind phase of trial	48	Almost no attacks Good QoL (clinician reported) Patient benefited from frequent contact with research nurses during trial and support with self-cannulation during the double-blind period of the trial, when acute treatment was required
9	Increased frequency and severity	None	1,000–1,500 U IV pdC1-INH twice/week	Dose of pdC1-INH titrated to 500 U QOD. Danazol reinstated once patient had ceased breastfeeding	NA	One to three attacks/year AE-QoL Questionnaire total score = 36.76
10	Increased frequency with mostly abdominal attacks	Fatigue	Icatibant 30 mg on-demand	On-demand C1-INH, prophylactic IV or SC C1-INH, and lanadelumab also available, but patient reinstated danazol after 19 weeks	NA	No attacks Patient has strong reservations about using injectables and a strong psychological dependence on danazol

AE-QoL, Angioedema Quality of Life; IV, intravenous; NA: not applicable; QD: every day; QOD: every other day; SC, subcutaneous

### Side effects of AA discontinuation

Seven patients had no side effects of discontinuation other than changes to HAE attack frequency and/or severity. Three patients experienced the following: anxiety and depression (likely due to developing cancer as well as changes to attack frequency; *n* = 1), weight gain (*n* = 1) and fatigue (*n* = 1).

### Patient outcomes after AA discontinuation

Of the 7 patients who remained off AA treatment, time since discontinuation ranged from 7 to 84 months (median = 48 months); the majority of patients went on to receive a different form of prophylaxis (n = 6), while 1 patient received only on-demand therapy. At data collection, these patients were experiencing no or very few HAE attacks, and quality of life (QoL) had largely improved. Of the 3 patients who restarted AAs, HAE attacks continued for 2 patients whose QoL was either moderately affected or described as ‘poor’. The third patient who reintroduced AAs has experienced no attacks, but remains anxious about introducing injectable treatments.

## Discussion and conclusions

Although AAs have been the historical option for long-term prophylaxis for HAE [6], there is now a shift away from these drugs [38, 39], which may continue as further targeted therapies are developed and approved, such as IV or SC pdC1-INH, lanadelumab and berotralstat [32–35, 37, 47, 48]. Our case series illustrates the heterogeneity of AA discontinuation strategies and the risk of increased HAE attack frequency and severity, alongside the potential positive outcomes for patients with HAE caused by C1-INH deficiency if appropriate management is instigated after AA withdrawal.

The most appropriate protocols for managing discontinuation of long-term AA prophylaxis have not yet been identified [40–43]; while tapering, overlapping and immediate withdrawal strategies have been suggested, these have not been systematically compared in terms of patient outcomes [45]. It is likely that a whole range of factors beyond attack control, such as patient requirements/preferences and the availability of other options, will play a role in finalizing discontinuation strategies.

The most common challenge when patients discontinued AAs was an increase in HAE attack frequency and/or severity; a challenge previously highlighted by the survey of physicians treating HAE [45]. It is important for patients and clinicians to be aware of the risk of increased attack frequency and severity, and prior to discontinuing androgens, patients should be made aware of the potential need to manage severe breakthrough attacks, and should have both access to therapies and confidence in administering these therapies promptly. HAE attack frequency and severity should be monitored closely, using either the Angioedema Activity Score [49], or patient reports or diaries, to ensure that the provided therapies continue to manage attacks. Disease registries can be particularly useful in the monitoring of disease evolution, especially if patients can autonomously enter their data in real time. Patients may require training or retraining in administering therapies because, in some cases, patients may be switching from oral AAs to injectable therapies or patients may not have had to manage a breakthrough attack for several years. Patient training must be clearly communicated, with continued support to ensure that care plans align with any changes in HAE attack frequency or severity, and patient needs [6]. Healthcare professionals should be ready to modify the treatment strategy at any stage if HAE attacks are not controlled, and follow-up appointments can be systematically included in treatment plans to ensure that any changes in attacks are communicated promptly.

Beyond the physical risk of HAE attacks, fatigue, anxiety and depression have been reported in patients with HAE discontinuing AAs [45]. Although AA discontinuation in patients with HAE has not been systematically studied, in our case series 7/10 patients reported no side effects other than changes to HAE attack frequency or severity.

The post-AA period can also herald a psychological burden, particularly in patients experiencing minimal HAE attacks while being treated with AAs, and patients require additional support to manage these anxieties. For some patients, attachment to oral AAs can be high, even in the presence of side effects, and these patients emphasize the need for education and support during and after discontinuation. One patient in our case series reintroduced AAs due to anxieties over the use of injectable on-demand and prophylactic therapies and a psychological attachment to the AAs that had controlled his attacks for 36 years. The reasons for patients not completing discontinuation or returning to AAs are also highly dependent on available resources and therapy types. As exemplified by another of the cases here, the return to AA treatment after a severe laryngeal attack can be based on limited availability of other options.

Although our case series was limited by size, the seven patients who discontinued AAs and resumed treatment with a different option experienced improvements in HAE attack control. One patient who continued treatment with on-demand therapy only has experienced only one HAE attack in a 7-year period. This emphasizes the need to regularly assess patients for prophylactic requirements. Although the management of HAE attacks is crucial, the potential impact of continuous treatment must also be considered.

In our study of real-world cases, the limited numbers of patients combined with heterogeneity of clinical circumstances and variable long-term AA regimens do not permit us to draw firm conclusions on the most appropriate strategies for AA withdrawal. It is clear that patients must be monitored closely for increases in HAE attack frequency and severity, but with careful planning and monitoring, and appropriate resources and support, discontinuation can be well managed. While approaches to AA discontinuation in HAE have been suggested [45], to develop the required understanding of and provide standardized guidance for AA discontinuation in HAE, systematic studies in higher numbers of patients are required.

Such studies should be extensive and involve national or international networks of HAE experts, such as the global network of Angioedema Centers of Reference and Excellence (ACARE) [50]. Indeed, the ACARE network recently initiated the SHAERPA (Stopping Androgen Treatment in Patients with HAE—Characterization of Reasons and Protocols and Development of Advice for Patients and Physicians) project with the aim of developing consensus guidance on how to discontinue AA treatment based on patient data. The SHAERPA project will provide a platform for the systematic studies required to support future clinicians when transitioning patients from AAs to targeted therapies.

Recommendations on how to discontinue AAs should include details not only on how to manage discontinuation and changes to HAE attacks but also on how to support patient monitoring and education in order to help clinicians when transitioning patients from AAs to targeted therapies.

In conclusion, discontinuation of AAs is already, and will continue to be, a major topic in HAE management because of side effects, contraindications for AAs, and the availability of better tolerated drugs. While small, our case series highlights the heterogeneity of managing AA withdrawal and the possible destabilization of HAE control, and how replacement therapies are needed to support AA withdrawal for the majority of patients. The ongoing SHAERPA study followed by data‑driven recommendations will support the management of AA discontinuation to improve QoL for HAE patients.

## Supplementary Information

**Additional file 1. **Word document.doc; Patient cases; Further details of attenuated androgen discontinuation in hereditary angioedema cases series.

## Data Availability

The dataset supporting the conclusions of this article is included within the article and its Additional file 1.

## Abbreviations

AA: Attenuated androgen
ACARE: Angioedema Centers of Reference and Excellence
AE-QoL: Angioedema Quality of Life
C1-INH: C1-inhibitor
HAE: Hereditary angioedema
IV: Intravenous
pd: Plasma-derived
QD: Every day
QOD: Every other day
QoL: Quality of life
rh: Recombinant human
SC: Subcutaneous
SHAERPA: Stopping Androgen Treatment in Patients with HAE—Characterization of Reasons and Protocols and Development of Advice for Patients and Physicians
SPC: Summary of product characteristics
U: Units
