# Supplementary material for: Attenuated androgen discontinuation in patients with hereditary angioedema: a commented case series
Source: Allergy Asthma Clin Immunol. 2022 Jan 13;18:4. doi: 10.1186/s13223-021-00644-0 (PMC8759255; doi:10.1186/s13223-021-00644-0)
Supplement: Supplementary file 1 — Additional file 1. Word document.doc; Patient cases; Further details of attenuated androgen discontinuation in hereditary angioedema cases series. [file 13223_2021_644_MOESM1_ESM.docx]

# Additional file 1

## Case 1

This 50-year-old female patient received danazol for 28 years prior to discontinuation. Dose reductions from 200 mg QD to 200 mg QOD had already been attempted in order to manage side effects (headaches, hypertension, muscle cramps, virilisation [hirsutism and vocal changes] and weight gain). These reductions resulted in an increased frequency and severity of breakthrough attacks, and the danazol dose was returned to 200 mg QD. When lanadelumab became available, the patient switched treatments from danazol 200 mg QD to lanadelumab 300 mg every 14 days, with no gradual reduction of dose or washout period. Icatibant 30 mg was provided to this patient for on-demand treatment of breakthrough attacks. No breakthrough attacks or side effects were experienced during treatment switch and no attacks have been experienced in the 10 months since discontinuation (Angioedema Activity Score = 28). The patient showed improvements in QoL (as assessed using AE-QoL), experienced a reduction in headaches and lost weight.

## Case 2

This 34-year-old male patient experienced recurrent abdominal attacks. Oxandrolone 5 mg QD was introduced for 8 months and then increased to 7.5 mg to manage abdominal attacks occurring approximately every 2 weeks. His abdominal attacks were controlled on this dose for 4 months, but he developed polycythaemia; hence, oxandrolone was paused for 3 months. Oxandrolone was reintroduced at 5 mg QD, but he continued to require rhC1-INH 12,600 U once/week for 3 months and attack control was poor, with both abdominal and peripheral attacks. pdC1-INH 2,000 U twice/week was introduced with on-demand icatibant, but breakthrough attacks continued at a rate of one to four attacks per month. Prophylaxis was increased to pdC1-INH 2,000 U three times/week, but the patient was eventually switched to lanadelumab 300 mg twice/month with on-demand pdC1-INH 2,000 U or icatibant 30 mg, and he has had no attacks in the 6-month period since the introduction of lanadelumab.

## Case 3

This 52-year-old male patient was treated with danazol 100 mg QD and had no attacks for 10 years. After a slight increase in attack frequency, danazol was increased to 200 mg QD. The patient began to gain weight and had headaches, hypertension and myalgia; he then transitioned to prophylaxis with pdC1-INH 1,000 U twice/week. At the same time, danazol was reduced from 200 mg QD to 100 mg QD for 2 weeks, 100 mg QOD for 2 weeks, and then 100 mg/week for 2 weeks, before being stopped. The patient had a series of severe abdominal attacks with rapid development (2 hours to attack peak) 9 days after reduction of danazol. All attacks required on-demand pdC1-INH but had reduced in frequency and severity by 2 months post-AAs. The patient also had a high weight gain during discontinuation, but this has since reduced, along with other side effects (headaches, hypertension and myalgia). His QoL has been good since he discontinued danazol 6 years ago.

## Case 4

This 76-year-old male patient was treated with danazol for 18 years, after being symptomatic for more than 30 years without any treatment. The basal attack rate before danazol initiation was one to two attacks per month. After starting on a dose of 100 mg QD, the patient’s attacks were fully controlled for 9 years until ramipril was introduced because of high blood pressure. The use of an angiotensin converting enzyme (ACE) inhibitor triggered HAE attacks and the dose of danazol was increased to 200 mg QD. When blood cholesterol levels increased, statins were prescribed (statins and danazol are both metabolized by CYP3A4 and concomitant administration increases the risk of rhabdomyolysis, particularly where high doses are used).[18] In 2013, the patient became symptomatic again and the dose of danazol was further increased to 300 mg QD. Shortly after this dose increase, the patient experienced rhabdomyolysis and renal impairment that required dialysis. AAs were immediately withdrawn and on-demand icatibant 30 mg was prescribed to manage further attacks. Only one attack has occurred over a 7-year period.

## Case 5

This 64-year-old female patient was treated with danazol at 600 mg QD, which was reduced to 150 mg QD when the patient started menopause. After 14 years, she was diagnosed with breast cancer and immediately stopped treatment with danazol because of contraindications with anti-cancer therapies (surgery, radiotherapy and exemestane). The patient was provided with icatibant 30 mg as an on-demand therapy, but attack frequency increased, with some abdominal attacks that relapsed despite prompt treatment. The patient experienced depression and anxiety after AA discontinuation, but these are likely associated with the progression of cancer and changes to hormone therapy, as well as the increased frequency of attacks. She was treated with antidepressants, and pdC1-INH 1,000 U every 3 days was introduced 6 months after discontinuation to manage the increased attack rate. Lanadelumab treatment at 300 mg every 14 days replaced pdC1-INH after the patient developed deep vein thrombosis (likely related to a new ovarian malignancy). Seven years after discontinuation, the patient is attack-free and satisfied with her prophylactic treatment, although she is concerned about the progression of her cancer.

## Case 6

This 31-year-old male patient was treated with danazol 200 mg QD and the frequency of attacks was greatly reduced. Danazol was then reduced by 200 mg/week every 6 months until the dose of 200 mg three times per week was reached, which was maintained for 3 years. This strategy was repeated to reach 200 mg twice per week, but abdominal attacks occurred and a dose of 200 mg three times per week was reintroduced. A further attempt to reduce the dose was made using the above strategy (reductions of 200 mg/week every 6 months) and the patient received 200 mg/week for 2 years. Abdominal attacks then increased in frequency and danazol was increased to 200 mg three times per week for 6 months. A further reduction was made to 200 mg twice per week, but abdominal attacks then reoccurred after 1 year, and doses of 200 mg four times per week and then five times per week were required. This dose increase still resulted in at least one severe abdominal attack per month, so the patient was switched to lanadelumab at 300 mg twice a month. Danazol at 200 mg five times per week was maintained for the first 2 weeks of lanadelumab treatment, and no attacks occurred within 7 months of treatment switch. The patient experienced no side effects of dose reductions or discontinuation. The patient is now attack-free and there are plans to reduce the dose of lanadelumab to 300 mg/month.

## Case 7

This 59-year-old male patient initiated danazol at 600 mg QD. The dose was titrated down to a minimal effective dose of 100 mg QD, via 400 mg QD and 200 mg QD. During this period, the patient experienced hypercholesterolaemia, transaminase elevations, steatosis and multifocal leukoencephalopathy, and AAs were eventually discontinued because of a reduction in the frequency and severity of attacks. After discontinuation, the patient was provided with icatibant 30 mg as the frequency of attacks increased. The patient experienced a severe laryngeal attack, and as he had exhausted his icatibant supply, the patient was admitted to an HAE referral centre via ambulance, where he experienced airway obstruction and respiratory failure. This emergency was resolved through cricothyrotomy and pdC1-INH 2,000 U. The patient was reintroduced to danazol 200 mg QD upon discharge from hospital, and this has since been titrated to 50 mg QD. His QoL remains poor and disease control is not optimal; the metabolic effects and multifocal leukoencephalopathy have not been resolved.

## Case 8

This 48-year-old male patient was initially provided with danazol as long-term prophylaxis, but this resulted in mood disturbances, so oxandrolone was introduced at 5 mg QD, and the dose varied between 5 mg and 7.5 mg, depending on attack frequency. Partial disease control was achieved with one or two (mostly peripheral) attacks/week and abdominal attacks every 1–2 months, some of which required hospital admission for several days. On-demand C1-INH was provided to manage breakthrough attacks. The patient discontinued oxandrolone to begin treatment with pdC1-INH 1,000 U twice/week, but returned to oxandrolone treatment because of poor efficacy and adherence. The patient successfully discontinued oxandrolone to join a clinical trial of lanadelumab. Although he required on-demand C1-INH to manage breakthrough attacks (one or two attacks per month) during the double-blind phase of the study, no further on-demand treatments have been required on open-label lanadelumab 300 mg every 14 days. Occasional mild attacks occur if lanadelumab is delayed beyond 2 weeks. His attacks have been almost fully controlled for 4 years. He has benefited from contact with research nurses and support and training for IV cannulation during the double-blind phase of the trial when he still had HAE attacks requiring treatment.

## Case 9

This 43-year-old female patient was treated with danazol for 29 years, with no dose reductions. Previous attempts at dose reductions resulted in increased attacks. Danazol at 100 mg QOD was discontinued immediately because of an unplanned pregnancy. Prophylactic treatment was commenced with pdC1-INH 1,000–1,500 U twice/week, but severe breakthrough attacks occurred, which required on-demand treatment with pdC1-INH 500 U. Prophylactic treatment was titrated to pdC1-INH 500 U  QOD, which prevented further attacks. After cessation of breastfeeding, the patient returned to long-term prophylaxis with danazol, combined with pdC1-INH for short-term prophylaxis and with pdC1-INH, rhC1-INH or icatibant for on-demand treatment. Mild attacks occur at a rate of one to three attacks per year, and the treating clinician plans to reintroduce a targeted therapy.

## Case 10

This 62-year-old male patient was treated with danazol for 36 years, with the starting dose of 600 mg QD reduced to an effective dose of 100 mg QD. Only one abdominal attack occurred during the period of AA treatment. The reason for discontinuation was loss of access to androgens, and the dose was tapered rather than immediately withdrawn because of the long period of time spent using AA prophylaxis. Danazol was reduced to 100 mg QOD for 1 week and then 100 mg every 3 days for 3 weeks. Icatibant 30 mg on-demand was introduced on the day that AA prophylaxis ceased, with patient education provided on self-administration.

The patient began to experience attacks up to once a week. These were mostly abdominal, but some affected the extremities and the genitals. After two months the patient reported fatigue. Long-term prophylactic options were discussed, but the patient had strong reservations about using these and a strong psychological attachment to danazol. A close monitoring strategy was agreed, but 19 weeks after discontinuing danazol the patient had reintroduced danazol prophylaxis at 100 mg QD via an international pharmacy and a prescription from his general practitioner; he has had no attacks since AAs were recontinued.
